# CaMKIV over-expression boosts cortical 4-7 Hz oscillations during learning and 1-4 Hz delta oscillations during sleep

**DOI:** 10.1186/1756-6606-3-16

**Published:** 2010-05-24

**Authors:** Hendrik W Steenland, Vincent Wu, Hotaka Fukushima, Satoshi Kida, Min Zhuo

**Affiliations:** 1 Department of Physiology, University of Toronto, Centre for the Study of Pain, 1 King's College Circle, Toronto, Ontario, M5S 1 A8, Canada; 2 Department of Bioscience, Faculty of Applied Bioscience, Tokyo University of Agriculture, Tokyo, 156-8502, Japan

## Abstract

Mounting evidence suggests that neural oscillations are related to the learning and consolidation of newly formed memory in the mammalian brain. Four to seven Hertz (4-7 Hz) oscillations in the prefrontal cortex are also postulated to be involved in learning and attention processes. Additionally, slow delta oscillations (1-4 Hz) have been proposed to be involved in memory consolidation or even synaptic down scaling during sleep. The molecular mechanisms which link learning-related oscillations during wakefulness to sleep-related oscillations remain unknown. We show that increasing the expression of calcium/calmodulin dependent protein kinase IV (CaMKIV), a key nucleic protein kinase, selectively enhances 4-7.5 Hz oscillation power during trace fear learning and slow delta oscillations during subsequent sleep. These oscillations were found to be boosted in response to the trace fear paradigm and are likely to be localized to regions of the prefrontal cortex. Correlation analyses demonstrate that a proportion of the variance in 4-7.5 Hz oscillations, during fear conditioning, could account for some degree of learning and subsequent memory formation, while changes in slow delta power did not share this predictive strength. Our data emphasize the role of CaMKIV in controlling learning and sleep-related oscillations and suggest that oscillatory activity during wakefulness may be a relevant predictor of subsequent memory consolidation.

## Background

There is evidence that these prefrontal 4-7.5 Hz rhythms, which exist in mice, rats, monkeys and humans, are involved in attention and learning processes [1-4]. In addition, human and rodent studies suggest that slow oscillations during sleep are related to memory [5-8]. Parallel studies examining single neuron recording in the prefrontal and hippocampal cortex show that there is a network of information replay during sleep, which reflects learning during the days experience [9-12]. However, there is also evidence for neural replay in the hippocampus during periods of wakefulness [13,14]. While studies have largely focused on the possibility of memory consolidation being related to neural oscillations during sleep, few studies have attempted to relate the neural oscillations during learning with that of subsequent sleep. Moreover, genetic manipulation may be helpful to connect neural oscillations which occur during learning with those that occur during sleep.

At the molecular level, gene transcription and new protein synthesis play critical roles in memory consolidation. Inhibition of protein translation processes prevent or inhibit memory consolidation [for reviews, see [15,16]]. The cAMP response element-binding protein (CREB) is a major activity-dependent transcriptional factor involved in the formation of long-term memory [17-19] [for reviews, see [20,21]]. Two major pathways responsible for CREB activation are the cAMP signaling pathway and the CaMKIV pathway [22,23] [for review, see [24]]. CaMKIV is a serine-threonine kinase and is activated by a combination of elevated intracellular Ca^2+ ^and CaMK kinase during neuronal activity [for review, see [25] and phosphorylates CREB [26,27]]. While the roles of CREB-related pathways in synaptic potentiation and initial learning are becoming clear, their possible contribution to memory consolidation and cortical slow delta oscillations during sleep has not been examined.

CaMKIV is involved in synaptic plasticity [28], synaptic homeostasis [29], learning and memory [22,30-33]. In addition, CaMKIV influences the CREB pathway [26,34], and its mRNA is up-regulated following sleep [35]. Thus, we hypothesized that CaMKIV over-expression will impact EEG oscillations associated with learning, memory and sleep. To test these hypotheses we used transgenic mice, over-expressing CaMKIV in the forebrain under the control of alpha CaMKII promoter [32,33].

## Methods

Transgenic mice were generated in the laboratory of Dr. Satoshi Kida, using a transgene that contained a αCaMKII promoter, hybrid intron in the 5' untranslated leader, the coding region of the CaMKIV fused with the Flag sequence at the N-terminus and a polyadenylation signal [32]. Frontal-parietal electrode implantation was conducted on 11 male CaMKIV over-expressed mice (average age = 12.3 weeks) and 11 male wild type (WT) control (4 C57Bl6 from Charles River and 7 cage-mate control (C57Bl6 background) (average age = 12.2 weeks)). Surgery was performed on 12 C57Bl6 mice from Charles River for intracortical field potential recording (average age = 9.9 weeks). Mice were maintained on a 12:12-h light-dark cycle (lights on 8:00 AM), and had access to food and water ad libitum. Procedures conformed to the recommendations of the Canadian Council on Animal Care and the University of Toronto Animal Care Committee approved the protocols.

### Surgical preparation

#### Frontal-parietal EEG

Mice were anesthetized with 1-2% isoflurane which was mixed with oxygen (30% balanced with nitrogen) and delivered to the mice via nose cone throughout the surgery. All electrodes were pre-attached to a miniature connector. The abdomen and scalp of mice were shaved and then cleaned with iodine (Triadine) and alcohol. The skull of the mouse was fixed into a stereotaxic adapter (502063, WPI, Sarasota, Fl, USA) mounted on a stereotaxic frame (Kopf Model 962, Tujunga, CA, USA). Three small holes (1.19 mm diameter) were drilled into the skull for differential frontal-parietal recordings. Electrodes, consisting of a wire attached to a jewelers screw (with contact end ground flat), were fixed into the holes to record EEG (electroencephalogram) at the following coordinates relative to bregma: frontal cortex (AP 2.2, ML 1.0), parietal cortex (AP -2.2, ML -2.5) and ground (AP -3.0, ML 3.0) [36]. Dorsal neck muscles were also exposed and Teflon-coated stainless steel electrodes (Cooner Wire) were sutured (4.0 silk) to each muscle to record neck EMG (electromyogram). The wires and connector were secured to the skull with dental cement and cyanoacrylate glue (Krazy glue). Mice were injected, intraoperatively (SC) with buprenorphine (0.1 mg/kg) as an analgesic, and 1.0 ml sterile saline (IP) for hydration. Mice were placed on a warm heating pad until they showed signs of ambulation, and were permitted to recover ~14 days prior to recording.

#### Cortical field recording

Intracortical EEG recording was performed with sterile bipolar Teflon coated tungsten electrodes (AM-systems, 796000, 76.2 m wire diameter) with a 0.5 mm tip offset. The electrode impedances were 0.1-0.3 MOhms and matched within ± 50 kOhms of each other to improve common source noise rejection. All impedances were measured with an impedance tester (BAK electronics Inc., model IMP2, Mount Airy, MD, USA). Surgery was performed with similar technique as above. However, in these experiments, 2 bipolar electrodes were implanted into the cortex. When the animal's skull was exposed, two holes were also drilled over frontal (0.1 AP, 0.6 ML relative to bregma) and motor cortex (0.1 AP, 0.6 ML). Bipolar electrodes were then lowered at a 45 degree angle into the anterior cingulate cortex (ACC) (-1.8 DV) and in the motor cortex (-1.0 DV) from the skull. Holes were drilled for a stainless steel ground screw (AP -3.0, -ML 3.0) and a support screw (AP 1.0, ML -1.0) to help secure the electrode assembly. The regions where the electrodes penetrated the brain were covered with a mixture of bone wax and mineral oil. The electrode assembly was then fixed to the skull with dental cement and Krazy glue. Neck electrodes were implanted and the animal was left to recover as above. For localization of 4-7.5 Hz rhythms in the frontal cortex, 4 electrodes were inserted at a 45 degree angle into the prelimbic region (AP 1.4). The electrodes tip separation was 0.3 mm for these recordings.

### Sleep recording

The mice were placed in a clear plastic container situated in a cubicle (ENV-017 M, Med Associates, St. Albans, VT, USA) in the evening before any experimentation. On the day of the experiment, between 7 and 8 AM, a lightweight cable was connected to the assembly on the animal's head. The signals were routed through a commutator (Crist Instruments, Hagerstown, MD, USA) and data collected continuously for 8-24 hours. Electrophysiological signals were amplified and filtered (Super-Z headstage amplifiers and BMA-400 amplifiers and filters, CWE Inc., Ardmore, PA, USA) as follows: cortical EEG 1000 × at 1-100 Hz and neck EMG 2000 × at 100-1000 Hz. Neck EMG recordings were smoothed (25 ms time constant) and rectified (Spike2 software, 1401 interface, CED (Cambridge Electronic Design) Ltd., Cambridge, UK). For intracortical field potential recording, data was collected with a unity gain headstage (made in house) and the signals were amplified by 1000 × and filtered from 1-100 Hz (differential amplifier DP-304, Warner Instruments, Hamden, CT, USA).

### Trace fear memory recordings

Trace fear conditioning was performed in an isolatedshock chamber (Med Associates, St. Albans, VT). The conditioned stimulus (CS) was a 80 dB white noise, delivered for 15s, and the unconditioned stimulus (US) was a 0.75 mA-scrambled foot-shock for 0.5s [33]. Mice are acclimated for 60s, and presented with ten CS-trace-US-ITI trials (trace of 30 or 15s, inter-trial interval (ITI) of 210 or 225s). One day after training, mice are acclimated for 60s and subjected to ten CS-ITI trials (ITI of 210 or 225s) in a novel chamber to test for trace fear memory [37]. Freezing is typically defined as the absence of movement with the exception of breathing. Conventional trace fear paradigms have been performed with animal during the day, when animals normally sleep. This was previously necessary because behavioral scoring and motion detection require light to determine freezing behavior. Thus, most studies have been carried out when the animal would be naturally sleeping. To circumvent this potential confound we utilized neck EMG recording to examine freezing behavior in the dark (0.2 lux) when mice are naturally awake [38]. The additional advantage of recording in dim light or the dark is that visual cues may be reduced. Frontal-parietal EEG or prelimbic field potentials were obtained from animals during the paradigm to determine if there were any global EEG or local field changes respectively.

### Histology

At the completion of each local field potential experiment, animals were sacrificed with 2% isoflurane. A DC current was applied between the recording electrodes and ground electrodes to produce a lesion. The brain was then removed and fixed in 10% formalin. Three days later, the brain was cryoprotected in a 30% sucrose solution until the brain was dehydrated. Relevant brain regions were sectioned in the coronal plane on a cryostat (CM 1850; Leica), at 40 μm, for verification of electrode lesion sites. Coronal cross-sections were then registered with the stereotaxic atlas of the mouse brain [36] and the position of the electrodes recorded.

### Analyses

Periods of active wakefulness, rapid-eye-movement (REM) and non-REM sleep were scored visually off-line and the scorer was blinded to the experimental condition. Active wakefulness was characterized by high frequency low voltage EEG and neck muscle movement (twitches) lasting for at least 30s. Abatement of muscle movement for 10s or more constituted the end of the active wakefulness bout. NREM sleep was characterized by high voltage slow wave activity, with no muscle movement, low muscle tone and lasting a minimum of 30s. Increases in EMG or change in EEG to wakefulness for 3s or more was considered arousal from sleep and terminated the NREM sleep bout. REM sleep was scored with the record of neck muscle atonia coinciding with high frequency low voltage EEG (mostly in the 4-13 Hz range) and lasting more than 30s. Arousal from REM sleep consisted of an increase in neck muscle EMG coinciding with a reduction of the typical REM 4-13 Hz power. The 30s time periods described above, while not conventional, were used to produce a conservative estimate of the time and frequency spent sleeping (we were not interested in micro-sleeps). While our 30s criteria is arbitrary, it is based our assumption that behavioral states need to be stable to clearly represent what we were interested in (i.e. powers, frequencies and durations of Active wakefulness, NREM and REM sleep). This should minimize type I errors (false positives) and produce a conservative estimation of the impact of CaMKIV over-expression.

Scripts for EEG analysis (Sudsa-version 2.2) were obtained from CED. Fast-Fourier transform was used to convert EEG waveforms into total power (μV^2^), which was then binned every 5s for the following frequency bands: δ1 (1-2 Hz), δ2 (2-4 Hz), θ1 (4-7.5 Hz), θ2 (7.5-13 Hz), β1 (13-20 Hz), β2 (20-30 Hz) and γ (30-100 Hz), similar to others [39,40]. While this binned analysis results in a decrease in resolution, it will by definition produce a conservative estimate of changes in brain wave frequencies. Statistical analyses were conducted on absolute power spectra rather than relative (or normalized) power spectra for two reasons. Firstly, the number of animals in each group was of sufficient size that the actual power values could be reported. Secondly, EEG frequencies recorded in higher ranges (above 7.5 Hz) were relatively similar between WT and CaMKIV over-expressed mice suggesting that the observation were not a result of differences in electrode positions or differing amounts of contact between the dura and electrode. Thus, normalization is only necessary if all the frequencies in the EEG were shifted in an upward or downward direction, due to inconsistency in recording. Neck EMG was quantified by integrating the area under the rectified and smoothed EMG waveform every 5s. For each animal, every sleep and wakefulness period was scored and quantified. All data were copied to a spreadsheet and sorted according to the sleep or wakefulness states. A grand average across each sleep and wakefulness state was then computed for each animal.

To examine slow delta oscillations, the power from the two slow wave bands (1-2 and 2-4) were summed for each file of data collected. A grand average of slow delta power was collected for each hour beginning at the lights on period (8AM) for a total of 8 hours. Slow delta oscillation power was calculated for baseline sleep and sleep following trace fear training. This analysis was used for frontal-parietal EEG recordings. Normalization was used for intracortical field potential recordings and was necessary since the magnitude of the voltage from the motor cortex across all frequencies was much larger than the ACC across the entire night during baseline. Thus, for the motor cortex to be used as an appropriate control, both ACC and motor cortex slow delta power were normalized. Normalization was calculated by dividing the slow delta power of each hour (before or after fear conditioning) by the average slow delta power of the baseline recording prior to conditioning.

### Statistics

The analyses performed for each statistical test are included in the text where appropriate. For all comparisons, differences were considered significant if the null hypothesis was rejected at p < 0.05 using a two-tailed test. Mixed factor (1 between and 1 within subject variable) repeated measures ANOVA was performed and followed by post-hoc comparisons with the Bonferroni corrected P to infer statistical significance. Analyses were conducted with SigmaStat (SPSS Inc., Chicago, IL, USA) and data were plotted with SigmaPlot (Systat Software, San Jose, CA, USA). In some cases, where statistical tests involved multiple post-hoc comparisons, only the ANOVA is reported in text with significance indicated in the figures. Pearson-product moment correlation was used to examine the relationship between brain waves and fear behavior. For this analysis, changes in fear behavior and changes in EEG power were measured relative to baseline (baseline is identified as: EMG-freezing before conditioning, 4-7.5 Hz power before conditioning, slow wave power from NREM sleep of each hour, on day one) and expressed as a percentage of that baseline to yield percentage change.

## Results

### CaMKIV over-expression impacts natural sleep

**Figure 1:**
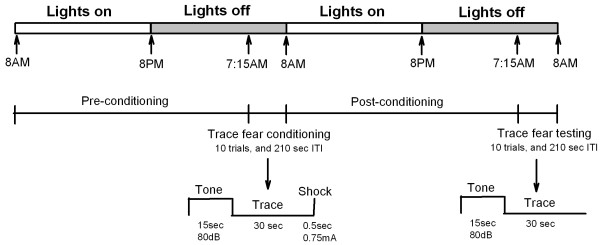
**Experimental design**. To examine behavioral states and freezing behavior, both WT and CaMKIV over-expressed mice were chronically instrumented with frontal-parietal EEG and EMG recording electrodes. Sleep and wakefulness recordings were obtained on the pre-conditioning day. Following the pre-conditioning day, between 7 and 8 AM, mice were trace fear conditioned. Mice were then returned to their home cage where their post-conditioning sleep was recorded. Trace fear conditioning was conducted for 45 minutes in a shock chamber. This paradigm involved repeated trials of a presentation of a tone, followed by an interval (trace interval) and subsequently by a foot shock. Trace fear testing was conducted the following day in a similar fashion, only in this case, the shock stimulus was omitted.

**Figure 2:**
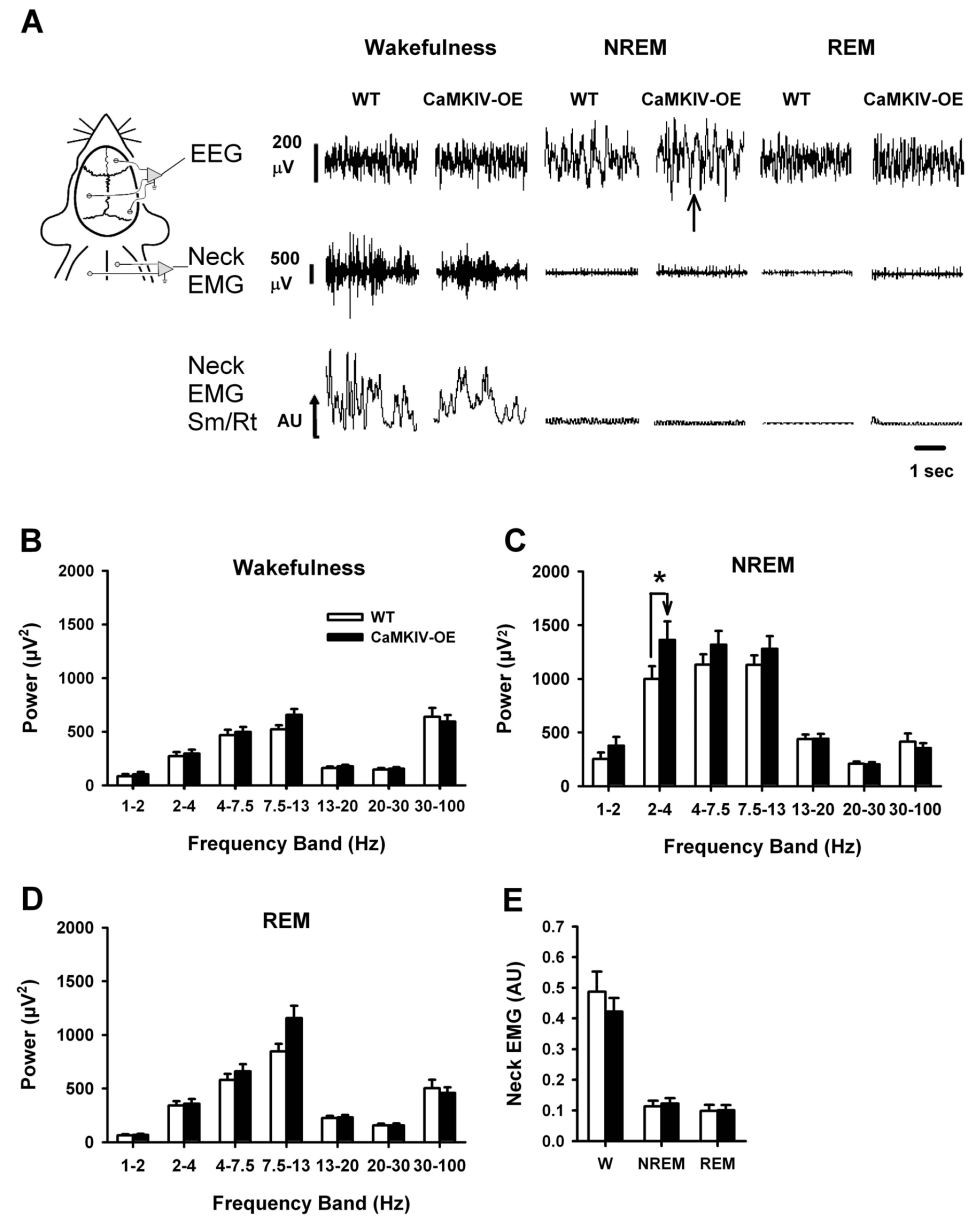
**CaMKIV over-expression enhances delta sleep in the recording environment**. (A) Example raw traces of sleep measurements in WT and CaMKIV over-expressed mice for wakefulness, NREM and REM sleep. To the left is a depiction of the location of the recording electrodes. CaMKIV over-expressedmice demonstrate relatively normal EEG during wakefulness and REM sleep but had elevated slow oscillations during NREM sleep (arrow). The raw neck and the smoothed and rectified (Sm/Rt) neck signals show that EMG activity in CaMKIV over-expressed mice was relatively normal across sleep and wakefulness states. (B) CaMKIV over-expressed mice have normal wakefulness EEG power compared to WT mice. (C) CaMKIV over-expressed mice have enhanced delta EEG power in the 2-4 Hz range compared to WT mice. (D) CaMKIV over-expressed mice have normal REM sleep EEG power compared to WT mice. (E) CaMKIV over-expressed mice have normal neck EMG across all behavioral states compared to WT mice. W is wakefulness, * indicate significant differences compared to wild type (WT) mice with *p *< 0.05, Values are means + SEM.

To examine whether CaMKIV over-expression alters sleep, learning and memory, we performed electroencephalogram (EEG) recordings with frontal-parietal electrodes according to the experimental design outlined in Figure 1. Figure 2A-D shows representative traces and grouped data of natural sleep and wakefulness states for CaMKIV over-expressed and WT mice. A significant interaction was detected between genetics and behavioral state for delta (2-4 Hz) power (*F*_(3,38) _= 2.83; *p *= 0.05, two-way ANOVA). Post-hoc analysis revealed that there was a significant increase in delta power selective to NREM sleep (*t *= 2.80; *p *= 0.008, Figure 2C). From Figure 2D, it also appears that there was an enhancement in 7.5-13 Hz power during REM sleep, however the ANOVA yielded neither a main effect (*F*_(1,20) _= 2.49; *p *= 0.124, two-way ANOVA) nor an interaction (*F*_(3,38) _= 1.46; *p *= 0.241, two-way ANOVA) for this frequency, between CaMKIV and WT mice.

Neck EMG activity was not different between CaMKIV over-expressed mice and WT mice (*F*_(1,20) _= 2.83; *p *= 0.644, two-way ANOVA), confirming that the sleep scoring was done consistently (Figure 2E). It was also found that the time spent in each particular sleep-wake state in the light (F_(1,6) _= 0.594, *p *= 0.470, two-way ANOVA) and dark (F_(1,6) _= 0.275, *p *= 0.630, two-way ANOVA) was not different between WT and CaMKIV over-expressed mice (Table 1). Finally, the frequency of occurrence of each particular sleep-wake state during the light (F_(1,6) _= 0.016, *p *= 0.904, two-way ANOVA) and dark period (F_(1,6) _= 0.001, *p *= 0.975, two-way ANOVA) was not different between WT and CaMKIV over-expressed mice (Table 1). Overall, these results show that CaMKIV over-expression augments delta-related EEG oscillations in the sleep recording environment without impacting the time spent sleeping or the frequency of sleep.

**Table 1:** CaMKIV over-expressed mice have normal sleep duration and sleep frequency. Values are means ± SEM

	WT	CaMKIV-OE	WT	CaMKIV-OE
*Lights On*	bouts/12 hrs	bouts/12 hrs	time/12 hrs	time/12 hrs
Wake	3.27 ± 0.51	3.75 ± 1.16	4.69 ± 0.87	4.94 ± 0.77
NREM	16.08 ± 1.68	15.21 ± 2.11	3.96 ± 0.73	4.04 ± 0.51
REM	2.38 ± 0.55	2.42 ± 0.26	0.60 ± 0.15	0.69 ± 0.08
*Lights Off*	bouts/11.25 hrs	bouts/11.25 hrs	time/11.25 hrs	time/11.25 hrs
Wake	3.78 0.41	4.44 ± 0.89	4.69 ± 0.87	4.94 ± 0.77
NREM	9.82 ± 1.97	8.93 ± 1.05	3.96 ± 0.73	4.04 ± 0.51
REM	1.11 ± 0.28	1.38 ± 0.23	0.60 ± 0.15	0.69 ± 0.08

### CaMKIV over-expression enhances learning and memory

**Figure 3:**
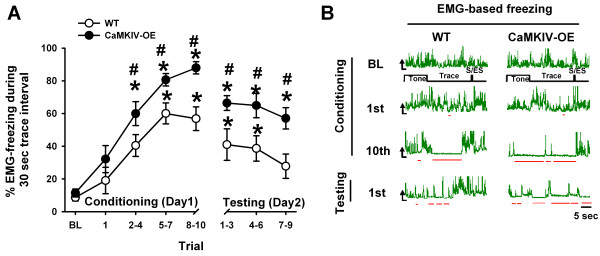
**CaMKIV over-expression enhances trace fear conditioning and subsequent memory**. (A) Trace fear conditioning (30s trace interval) learning curve for both WT and CaMKIV over-expressed mice (* is *p *< 0.05 compared to baseline (BL)). CaMKIV over-expressed mice have enhanced fear behaviors during trace fear conditioning and testing above that of WT mice (# is *p *< 0.05). (B) Example of EMG-based freezing and scoring (red lines under the trace) from WT and CaMKIV over-expressed mice. Raw EMG data show periods of quiescence during the conditioning and memory trials of the mice. During conditioning and memory testing, CaMKIV over-expressed mice demonstrate more EMG-based freezing behavior than WT mice. S/ES represents shock and expected shock, for the conditioning and test trials respectively, AU is arbitrary units.

CaMKIV over-expressed mice exhibit learning and memory enhancements for the trace fear paradigm [33]. In the present set of trace fear experiments, animals were trained and tested in the dark while EMG and EEG were simultaneously recorded. An EMG-based method, previously developed in our laboratory, was used to score freezing behavior [38]. This EMG recording method permits the measure of freezing in the dark phase, when nocturnal rodents are normally active. By having mice trained at this time, mice do not need to be disturbed during the light phase (i.e. sleeping phase) for training or testing, as is usually done in learning experiments. Secondly, this method, in conjunction with EEG can help discriminate sleep from fear behavior [38], and help minimize contextual visual information which could confound our study. Brain wave and freezing behavior measures were compared to the first 60s baseline taken in the recording chamber during trace fear conditioning. Other comparisons were made between WT and CaMKIV over-expressed mice. Figures 3A and 3B show summarized and raw EMG-based freezing data for WT and CaMKIV over-expressed mice during conditioning and testing days. There was significant effect of time interval (*F*_(7,140) _= 24.25; *p *< 0.001, two-way ANOVA), with both WT and CaMKIV over-expressed mice (Figure 3A) showing learning and memory retention. There was a significant effect of the genetic manipulation on freezing behavior (*F*_(1,20) _= 24.58 *p *< 0.001, two-way ANOVA), with CaMKIV over-expressed mice showing more freezing during both the conditioning phase and the testing phase. Thus, CaMKIV over-expressed mice learn and remember better then WT controls.

### CaMKIV over-expression and EEG responses to tone, trace interval, and shock

**Figure 4:**
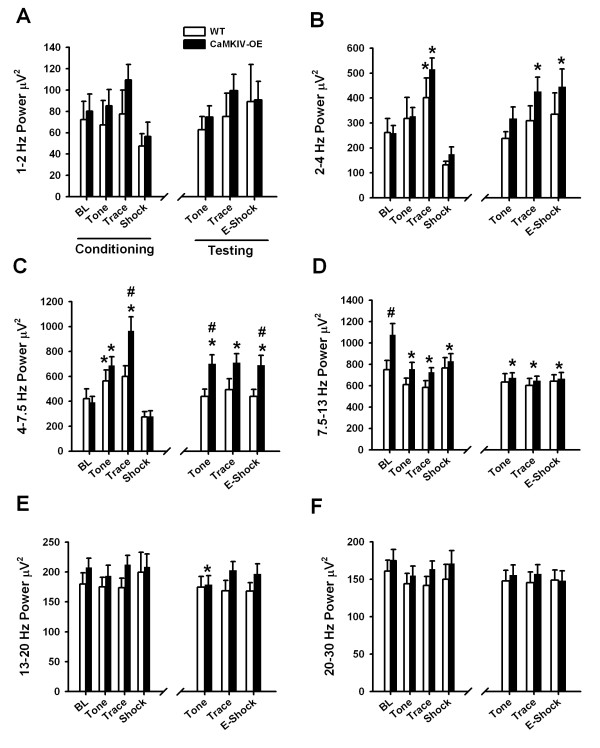
**CaMKIV over-expression and EEG responses to tone, trace interval and pain**. CaMKIV over-expressed mice demonstrate alterations in EEG rhythms during different phases of trace fear conditioning. EEG data was averaged across every trial during trace fear conditioning (left side of graph) and again for every trial during memory testing (right side of graph) for WT and CaMKIV over-expressed mice. (A) None of the interventions had a significant effect on 1-2 Hz EEG power. (B) Trace fear training increased 2-4 Hz power in both WT and CaMKIV over-expressed mice above baseline (* is *p *< 0.05) for the trace interval. (C) Trace fear conditioning significantly enhanced 4-7.5 Hz EEG power in CaMKIV over-expressed mice for both the tone and the trace interval conditions compared to baseline. WT mice only demonstrated an enhancement in 4-7.5 Hz EEG power for the tone interval (* is *p *< 0.05). In addition, there was a significant enhancement in 4-7.5 Hz EEG power of CaMKIV over-expressed mice above that of WT mice for the trace interval during conditioning and the expected shock (E-Shock) and tone interval during memory testing (# is *p *< 0.05 compared to WT). (D) The baseline 7.5-13 Hz EEG power was increased in CaMKIV over-expressed mice with initial placement into the fear conditioning chamber (# is *p *< 0.05 compared to WT) and reduced during the tone, trace and E-shock intervals (* is *p *< 0.05 compared to baseline). (E) 13-20 Hz EEG power was reduced during the tone presentation on the test day relative to baseline in CaMKIV over-expressed mice. (F) None of the interventions had a significant effect on 20-30 Hz EEG power. BL is baseline, and values are means + SEM.

There are numerous reports of brain wave oscillations associated with learning, memory, and attention in a variety of behavioral paradigms [41-43] [for review, see [44]]. We investigated the EEG responses for CaMKIV and WT mice to tone and trace intervals, and following a shock to the foot during the trace fear conditioning day (Figure 4A-F). We also examined the EEG responses during the testing day. However in this case, a shock was not delivered and so we examined the EEG of an expected (or anticipated) shock.

### EEG responses to tone

It is possible that CaMKIV over-expressed mice demonstrate enhanced arousal or attention to sound stimuli during conditioning. To investigate this, we analyzed EEG during the presentation of the tone. Mice were found to have normal EEG power, for most frequencies, during the presentation of the tone (Figure 4). However, there was a significant effect of the tone on 4-7.5 Hz power (*F*_(2,39) _= 20.89; *p *< 0.001, two-way ANOVA) and a significant interaction between genetics and the tone presentation (*F*_(2,39) _= 7.64; *p *< 0.002, two-way ANOVA). Post-hoc analysis revealed that the presentation of tone during conditioning enhanced 4-7.5 Hz power in CaMKIV over-expressed mice (*t *= 6.31; *p *< 0.001) and WT mice (*t *= 2.76; *p *< 0.026). When the tone was played on the testing day, post-hoc analysis revealed that 4-7.5 Hz power was increased for CaMKIV mice (*t *= 6.73; *p *< 0.001) but not WT mice (*t *= 0.331; *p *= 1.0) compared to their respective baseline. Finally, CaMKIV mice were found to have greater 4-7.5 Hz power during the tone on the testing day when compared to WT mice (*t *= 6.04; *p *< 0.001).

There was a significant alteration of 7.5-13 Hz power during the tone presentation (*F*_(2,39) _= 24.32; *p *< 0.001, two-way ANOVA) and a significant interaction between genetics and tone presentation (*F*_(2,39) _= 5.79 *p *< 0.006, two-way ANOVA). Post-hoc analysis revealed that, for CaMKIV over-expressed mice, presentation of a tone decreased 7.5-13 Hz power during both conditioning (*t *= 6.06; *p *< 0.001) and testing days (*t *= 6.92; *p *< 0.001) compared to baseline (Figure 4D). Additionally, CaMKIV over-expressed mice had elevated 7.5-13 Hz power above that of WT mice (*t *= 3.04; *p *< 0.005) during baseline. There was also a significant alteration of 13-20 Hz power during the tone presentation (*F*_(2,39) _= 4.38; *p *< 0.019, two-way ANOVA). Post-hoc analysis revealed CaMKIV over-expressed mice had reduced power during tone presentation on trace fear testing days (*t *= 3.64; *p *< 0.002) compared to baseline (Figure 4E).

### EEG responses during the trace interval

For a fear response to be conditioned across the trace interval, information about the tone must be retained until the shock is perceived. Since the hippocampus, in conjunction with the prefrontal cortex, is thought to maintain contiguity of fear memory during the trace interval [45,46] we examined whether or not there would be neural oscillations which corresponded to the trace interval. There was a significant effect of the trace interval on 2-4 Hz delta power (*F*_(2,39) _= 18.89; *p *< 0.001, two-way ANOVA). Post-hoc analysis revealed that there was a significant increase in 2-4 Hz power for both WT (*t *= 2.84; *p *= 0.016) and CaMKIV over-expressed mice (*t *= 5.89; *p *< 0.001) relative to baseline on the conditioning day (Figure 4B). For the testing day, CaMKIV over-expressed mice had significantly elevated 2-4 Hz power (*t *= 3.40; *p *< 0.005) compared to baseline. Overall, there was no effect of genetic manipulation on 2-4 Hz power, so this frequency is unlikely to explain the learning and memory enhancement in CaMKIV over-expressed mice.

There was a significant effect of trace interval on 4-7.5 Hz power (*F*_(2,39) _= 21.45; *p *< 0.001, two-way ANOVA) and a significant interaction between genetics and the trace interval (*F*_(2,39) _= 5.99; *p *= 0.005, two-way ANOVA-RM). Post-hoc analysis revealed that there was a significant increase in 4-7.5 Hz power for CaMKIV mice above baseline (*t *= 7.42; *p *< 0.001) for the conditioning day and above the baseline for the testing day (*t *= 3.73; *p *< 0.002, Figure 4C). In addition, CaMKIV over-expressed mice had a significant enhancement in 4-7.5 Hz power over WT mice during the conditioning day (*t *= 3.10; *p *= 0.004). These findings are consistent with the enhanced learning and memory of the CaMKIV mice (Figure 3) and may serve as a possible mechanism of attention processing, memory encoding or recall.

A significant alteration in 7.5-13 Hz power was detected across the conditioning interval (*F*_(2,39) _= 23.82; *p *< 0.001, two-way ANOVA). In addition, a significant interaction was detected between genetics and trace interval (*F*_(2,39) _= 4.46; *p *< 0.018, two-way ANOVA). Post-hoc analysis revealed that 7.5-13 Hz power was decreased in CaMKIV over-expressed mice compared to baseline for the conditioning (*t *= 5.78; *p *< 0.001) and the testing day (*t *= 6.58; *p *< 0.001, Figure 4D).

### EEG responses to shock and expected shock

Our previous studies have shown that CaMKIV over-expressed mice have normal responses to acute pain [33]. It is conceivable that CaMKIV mice have different levels of cortical arousal in response to foot shock which might be detected with EEG. This might provide a possible explanation for enhanced learning and memory in these animals in the trace fear paradigm. Thus, we analyzed whether or not EEG arousal responses to the foot shock differed between animals (Figures 4A-F). A significant alteration in 2-4 Hz power was detected for the shock condition (*F*_(2,39) _= 15.69; *p *< 0.001, two-way ANOVA). Post-hoc analysis revealed that 2-4 Hz power was increased in CaMKIV over-expressed mice (*t *= 3.12; *p *< 0.010) during the expected shock test condition, compared to baseline (Figure 4B).

There was a significant alteration of 4-7.5 Hz power following the shock stimuli (*F*_(2,39) _= 15.88; *p *< 0.001, two-way ANOVA) and a significant interaction with the genetic manipulation (*F*_(2,39) _= 4.17; *p *< 0.023, two-way ANOVA). Post-hoc analysis revealed an enhancement in 4-7.5 Hz power (*t *= 4.16; *p *= 0.001) during the expected shock condition, compared to baseline (Figure 4C). In addition post-hoc analysis revealed that CaMKIV over-expressed mice had significantly greater 4-7.5 Hz power during the expected shock condition (*F*_(2,39) _= 2.71; *p *< 0.009, two-way ANOVA). The increase of 4-7.5 Hz power appeared to be a result of the freezing behavior which carried over into the expected shock interval.

A significant alteration in 7.5-13 Hz power was detected during the expected shock interval (*F*_(2,39) _= 13.17; *p *< 0.001, two-way ANOVA). Post-hoc analysis revealed that 7.5-13 Hz power was decreased in CaMKIV over-expressed mice compared to baseline (*t *= 3.92; *p *= 0.001) during shock, but was unaltered in WT mice (*t *= 0.22; *p *= 1.00, Figure 4D). However, when WT mice were compared directly to CaMKIV over-expressed mice, no significant difference was detected (*t *= 0.55 *p *= 0.583), suggesting that their actual 7.5-13 Hz EEG power responses to the shocks are not likely to account for the enhanced learning and memory of CaMKIV mice. In conclusion, based on our previous findings [33] and the current EEG responses, we suggest that CaMKIV over-expressed and WT mice respond similarly to pain.

### Four-7.5 Hz oscillation enhancements parallel learning and can be localized to the prelimbic cortex

**Figure 5:**
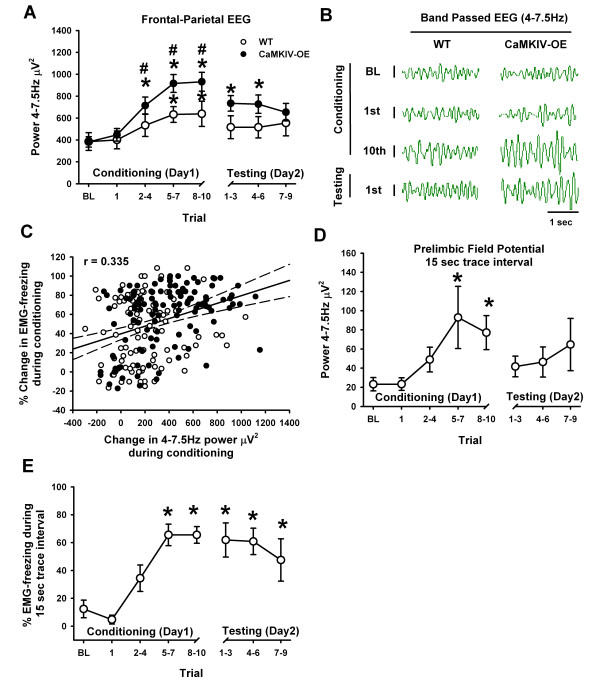
**CaMKIV over-expression enhances 4-7.5 Hz waves which can localize to the prelimbic cortex**. (A) Grouped data showing that WT and CaMKIV over-expressed mice have significant enhancements in 4-7.5 Hz power, during the trace fear interval, throughout the conditioning protocol (* is *p *< 0.05 compared to baseline). This effect mirrored the learning-curve for both mice. However, the magnitude of the effect was significantly greater in CaMKIV over-expressed mice as compared to WT for the conditioning phase (# is *p *< 0.05). (B) Example EEG activity corresponding to panel A, which was band-pass filtered for visual inspection of 4-7.5 Hz power. Four-7.5 Hz activity (50 μV scale bar) occurred during periods of freezing behavior and appeared to be more frequent and often larger in CaMKIV over-expressed mice. (C) Trial by trial changes in freezing during the trace interval were positively correlated to changes in 4-7.5 Hz power during conditioning. Dashed lines are 95% confidence intervals. (D) When WT mice are trained with a 15s trace fear interval, prominent 4-7.5 Hz power can be localized to the prelimbic cortex, during conditioning (* is *p *< 0.05 compared to baseline) but not on the testing day. (E) Trace fear conditioning (15s trace interval) learning curve for WT mice (* is *p *< 0.05 compared to baseline). Notice the memory enhancement when compared to Fig. 3A. BL is baseline, and values are means +/- SEM.

Since 4-7.5 Hz oscillations are enhanced in CaMKIV mice during learning and memory, above that of baseline and WT mice (Figure 4), we expected that these oscillations might represent neural correlates of attention or memory of the expected shock during the trace interval. We therefore expanded our 4-7.5 Hz power analysis to examine its time course as the mice learned. Figure 5A and 5B depict the 4-7.5 Hz activity across intervals of trace fear conditioning and testing. There was a significant main effect of the conditioning interval on 4-7.5 Hz power (*F*_(7,136) _= 17.28; *p *< 0.001, two-way ANOVA). For CaMKIV over-expressed mice, 4-7.5 Hz power was increased above baseline and exceeded that of WT mice (Figure 5A), producing a learning curve during conditioning. There was neither a main effect of genetics (*F*_(1,20) _= 2.994; *p *= 0.099, two-way ANOVA) nor or interaction between genetics and trace fear interval (*F*_(7,136) _= 1.97; *p *= 0.064, two-way ANOVA). Since these tests were nearly significant and an interaction between genetics and trace conditioning was found in Figure 4C, we ran post-hoc tests to see whether 4-7.5 Hz learning curves were different between CaMKIV and WT animals. Four-7.5 Hz power was also significantly elevated in CaMKIV over-expressed mice above baseline during the testing phase (~24 hours later) (*p *< 0.05, as in Figure 5A). The increase in 4-7.5 Hz power appeared to be related to a combination of increased voltage (Figure 5B) and the number of occurrences of this frequency during the trace interval. For WT mice, the 4-7.5 Hz EEG power increased during the conditioning but not the testing phase. This is the first evidence to show that CaMKIV manipulation can enhance, in a parallel fashion, the expression of fear behavior and 4-7.5 Hz EEG power.

To examine whether changes in 4-7.5 Hz power were related to changes in freezing behavior during the trace interval; the net change in 4-7.5 Hz power from baseline, for every animal of every trial, was quantified, plotted, and correlated against the change in freezing behavior from baseline for the respective trace fear interval. A significant correlation was found between the change in trace fear learning and changes in 4-7.5 Hz power (*r*^2 ^= 0.112, *p *< 0.001, Figure 5C). The results indicate that variation in freezing behavior (albeit a small amount) is related to variation in 4-7.5 Hz power.

Since 4-7.5 Hz frontal-parietal EEG power was increased during the trace fear interval, we wanted to identify if this frequency band can to be localized to a particular brain structure. Therefore, we chose to accentuate trace fear learning (analogous to the effect of CaMKIV over expression) by reducing the duration of the trace fear interval in order to make the time between shock and tone shorter. Since the prelimbic cortex is known to generate both theta rhythms [3,47,48] and be involved in trace fear conditioning [49,50] we placed intracortical electrodes in this region. Prelimbic field potential recordings were performed in WT mice under a 15 second trace interval protocol to localize 4-7.5 Hz rhythms. There was a significant effect of conditioning on 4-7.5 Hz activity recorded in the prelimbic cortex (*F*_(7,29) _= 3.64; *p *< 0.006, one-way ANOVA). Post-hoc analysis revealed significant enhancement of 4-7.5 Hz rhythms during trace fear conditioning (Figure 5D). Consistently, there was a significant effect of conditioning on fear behavior (*F*_(7,35) _= 13.61; *p *< 0.001, one-way ANOVA). Post-hoc analysis revealed significant learning and memory above baseline (Figure 5E). Thus when trace fear memory is accentuated in WT mice by making the trace interval shorter, enhanced 4-7.5 Hz rhythms can be localized to the prelimbic cortex during conditioning. Thus, it is likely that the enhancements seen in CaMKIV over-expressed mice with frontal-parietal EEG recordings would also localize to the prelimbic cortex. Moreover, our results show that accentuation of trace fear learning and memory, whether by CaMKIV over-expression or through manipulation of the trace fear interval, produces prominent enhancement in 4-7.5 Hz power. The finding that enhancement in prelimbic 4-7.5 Hz rhythms occurs during conditioning (15s protocol), but not testing contrasts with the finding that CaMKIV show enhancements during both conditioning (30s protocol) and testing. The results suggest that there may be additional structures, for example the hippocampus, which are activated during trace conditioning and memory recall and contribute to the more global EEG recordings.

### CaMKIV over-expression impacts slow delta oscillations

**Figure 6:**
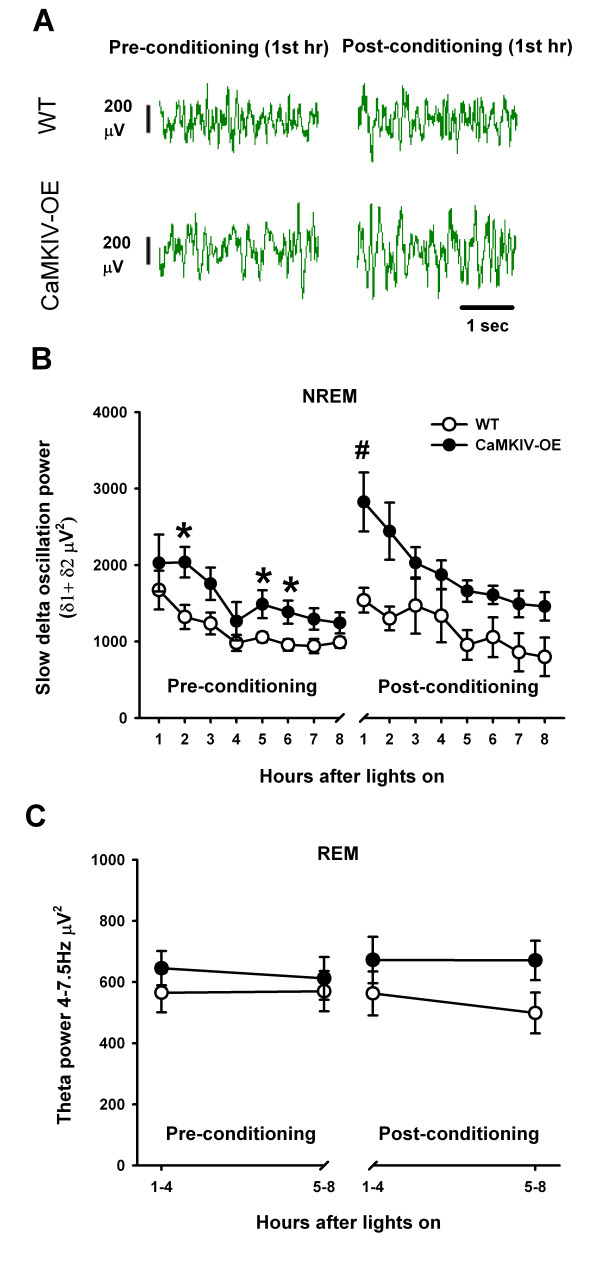
**CaMKIV over-expression enhances slow delta oscillation power during NREM sleep before and after trace fear training**. (A) Example of EEG sleep recording in the first hour, after the lights had been turned on, in the recording chamber for both CaMKIV over-expressed mice and WT mice. However, if the mouse had been trace fear conditioned, slow delta oscillation (1-4 Hz) enhancements were detected in the recording for CaMKIV over-expressedmice but not WT mice. (B) Group data showing that CaMKIV over-expressed mice have elevated slow delta oscillation power above that of WT mice during NREM sleep in the recording chamber across an 8 hour span. A subset of the CaMKIV over-expressed and WT mice were then recorded after trace fear training during the same time points of the next day. CaMKIV over-expressed mice were found to have elevated slow delta oscillations above that of the first hour of their pre-conditioning. However, WT mice did not show a similar enhancement. * is *p *< 0.05 compared to WT for the corresponding hour, # is *p *< 0.05 between pre and post-conditioning for the corresponding hour. (C) CaMKIV over-expressed and WT mice had similar 4-7.5 Hz power during REM sleep prior to and following trace fear conditioning. Four-7.5 HZ power was averaged across 4 hour time periods.

Since sleep in the recording environment increased 2-4 Hz EEG power, we decided to examine the time course of these slow delta oscillations (1-4 Hz) across the entire recording session. Slow delta oscillations are thought to be related to sleep need [51] and are potentiated by learning paradigms [8]. Slow delta oscillation power was averaged for each hour starting when the lights were turned on, for pre- and post-conditioning NREM sleep. For this analysis, separate statistical comparisons were made directly between WT and CaMKIV over-expressed mice for each hour. There was a significant effect of the genetic manipulation for the second (*F*_(1,23) _= 8.64; *p *= 0.007, two-way ANOVA), fifth (*F*_(1,22) _= 9.52; *p *= 0.005, two-way ANOVA) and sixth hour (*F*_(1,22) _= 6.05; *p *= 0.021, two-way ANOVA) (Figs. 6A and 6B). Further analyses revealed that CaMKIV over-expression elevated slow delta oscillation power above WT mice during the pre-conditioning phase for the second (*t *= 2.12; *p *= 0.034), fifth (*t *= 2.94; *p *= 0.007), sixth hour (*t *= 2.59; *p *= 0.015). This effect may be due to the novelty to the recording environment or a selective enhancement in brain waves that occurs during natural sleep.

We next examined the effect of trace fear conditioning on post-conditioning NREM sleep. For this analysis, separate statistical comparisons were made directly between WT and CaMKIV over-expressed mice for each hour. In addition we made separate comparisons between pre- and post-conditioning for each hour to examine interactions. While a similar trend for elevated slow waves in CaMKIV over-expressed mice above that of WT was seen, following trace fear conditioning, the trend did not achieve significance. However, a significant interaction was detected for the first hour comparisons, showing that the level of slow delta oscillation power depended, not only on whether the animal was trace fear trained but also on the genetic manipulation (*F*_(1,11) _= 5.1; *p *= 0.045, two-way ANOVA). Further analysis revealed that CaMKIV over-expressed mice have elevated slow delta oscillation power when compared to their preceding pre-conditioning baseline for the first hour (t = 3.99; *p *= 0.002). By contrast WT mice did not show the same enhancement (t = 0.22; *p *= 0.827). This result demonstrates that genetic enhancement of learning and memory (Figure 3A) also potentiates EEG activity during subsequent sleep in CaMKIV over-expressed mice, linking genetics, sleep and memory through the CaMKIV protein.

It was also examined whether REM sleep 4-7.5 Hz power would be influenced following trace fear conditioning. In contrast to, the slow delta power analysis in which 8 bins were used, REM sleep was broken down into two bins (1-4 and 4-8 hours post-conditioning). This was done because of the reliability of recording REM sleep for each individual hour was low. In contrast to the enhancements in slow delta oscillations during NREM sleep for CaMKIV over-expressed mice, we found no effect of genetic manipulation on REM sleep 4-7.5 Hz power before and after training within the first four hours (*F*_(1,20) _= 1.07; *p *= 0.312, two-way ANOVA) and last four hours (*F*_(1,20) _= 1.00; *p *= 0.330, two-way ANOVA) (Figure 6C).

**Figure 7:**
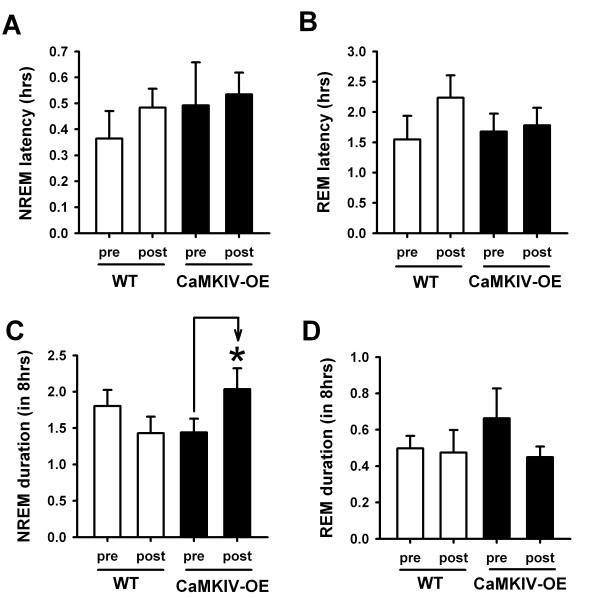
**Impact of trace fear conditioning on sleep duration and latency**. (A) The latency to enter NREM sleep before (pre) and after (post) trace fear conditioning was unaltered in CaMKIV over-expressed and WT mice. (B) The latency to enter REM sleep before and after trace fear conditioning was unaltered in CaMKIV over-expressed and WT mice. (C) WT mice showed a trend for decreased NREM sleep following trace fear conditioning, while CaMKIV over-expressed mice showed a significant increase in NREM sleep duration. * is *p *< 0.05. (D) The duration of REM sleep before and after trace fear conditioning was unaltered in WT and CaMKIV over-expressed mice.

It is possible that the increase in delta power in CaMKIV over-expressed mice may be secondary to an increase in the latency of falling asleep, following fear conditioning. There was no significant effect of genetic manipulation on the latency to NREM sleep (*F*_(1,14) _= 0.775; *p *= 0.393, two-way ANOVA), and REM sleep (*F*_(1,14) _= 0.331; *p *= 0.574, two-way ANOVA) following trace fear conditioning (Figures 7A and 7B). It is also possible that CaMKIV over-expressed mice may have increased slow delta power because their sleep is consolidated into a few bouts of very deep sleep, following trace conditioning. Interestingly, we found a statistically significant interaction between genetics and NREM durations before and after trace fear training (*F*_(1,13) _= 6.86; *p *= 0.021, two-way ANOVA). In contrast to our prediction, Post-hoc analysis revealed that CaMKIV over-expressed mice had more NREM sleep following trace fear conditioning (*t *= 2.23; *p *= 0.044, Figure 7C). In WT mice, NREM durations were not influenced by trace fear conditioning (*t *= 1.56; *p *= 0.142). Additionally, REM sleep durations were not significantly different (*F*_(1,14) _= 0.244; *p *= 0.630, two-way ANOVA) between WT and CaMKIV over-expressed mice (Figure 7D). Thus, the enhancement in slow delta oscillation power after trace fear training cannot be accounted for by alterations in sleep latency. The enhancement in delta power of CaMKIV over-expressed mice is further corroborated by the enhancement in time spent sleeping following trace fear conditioning. Indeed, the increase in NREM sleep duration in CaMKIV over-expressed mice reinforces the concept that genetics, sleep and memory may be linked through the CaMKIV protein. Thus, based on the findings we expect that slow wave enhancements following trace fear training may be related to learning.

### Slow delta oscillation enhancements are localized to the anterior cingulate cortex

Frontal-parietal EEG recordings yield a selective enhancement in 1-4 Hz slow delta power following trace fear conditioning for CaMKIV over-expressed but not WT mice (Figure 6). Thus, we needed to examine whether WT mice can also demonstrate enhanced delta power following trace fear conditioning. We chose to accentuate trace fear learning (analogous to the effect of CaMKIV over-expression) in WT mice by reducing the duration of the trace fear interval, in expectation that this would accentuate slow delta power during subsequent NREM sleep. Provided WT actually demonstrate enhanced delta power, we wanted to know whether it would localize to a particular brain region. c-fos is up-regulated in the ACC following trace fear conditioning without any effect in the motor cortex [52]. These results suggest that the ACC is involved in learning and memory processes associated with trace fear conditioning. Thus, we examined whether slow delta oscillations would be potentiated in the ACC following trace-fear conditioning, indicative of memory consolidation.

**Figure 8:**
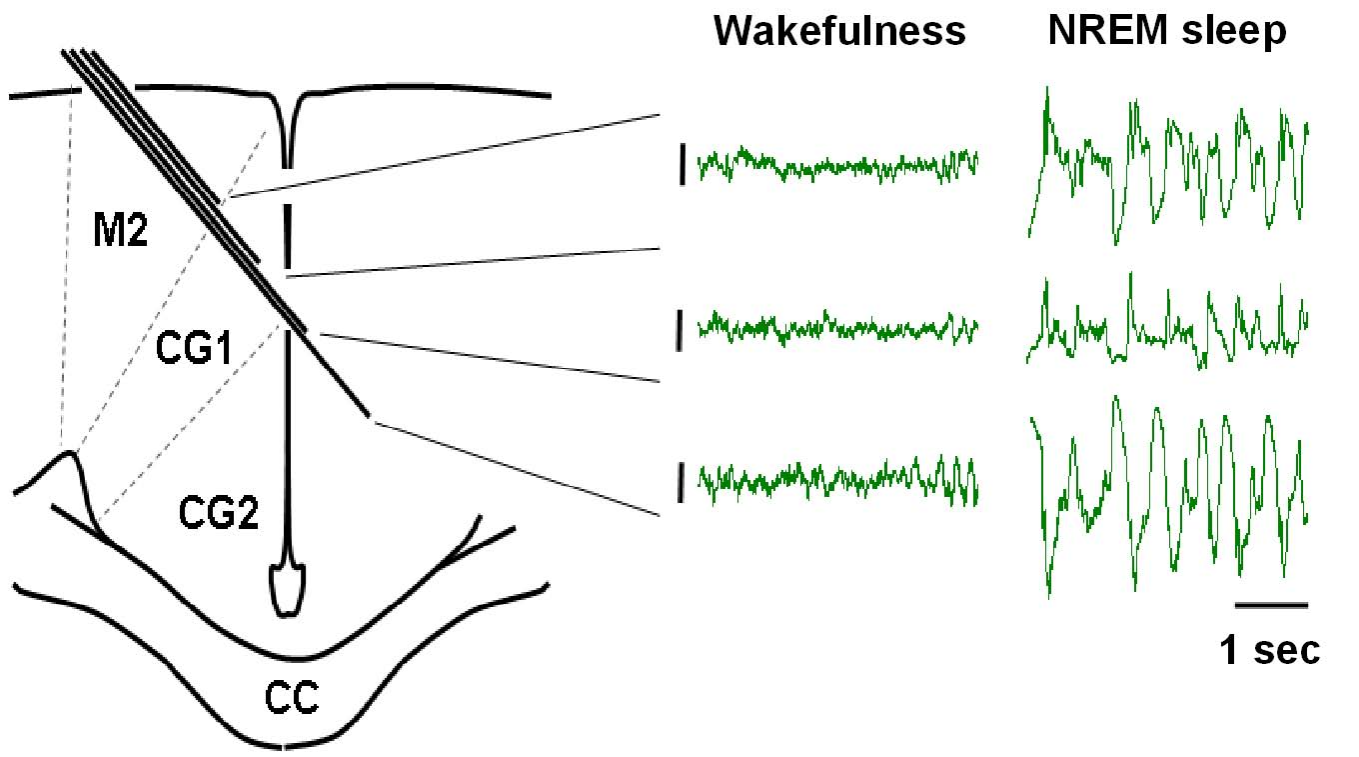
**Electrode positioning for ACC experiments**. Four electrodes were implanted into the ACC. Differential recordings were performed between adjacent electrodes. Field potential voltage is most robust when straddling layers I-III of the ACC. The polarity of the recordings is opposite for the upper trace and lower trace since the electrode crosses the midline. The differences in polarity are most evident in NREM sleep. Bars indicate 200 μV calibration.

**Figure 9:**
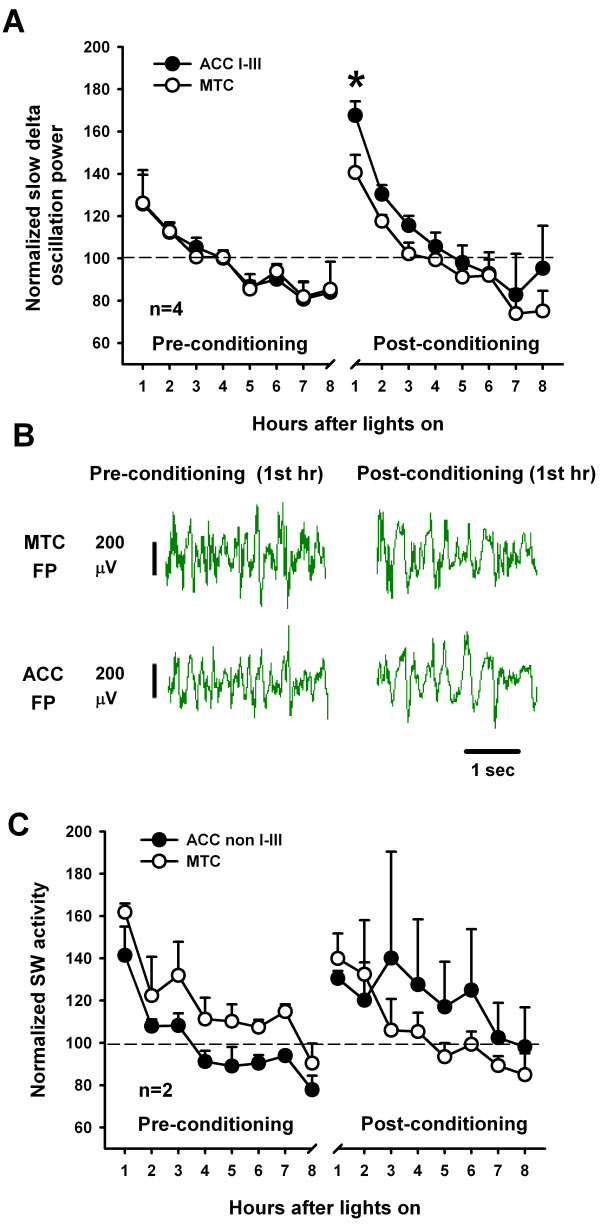
**Trace fear conditioning increases slow delta oscillations in the ACC in layer I-III**. (A) Group data showing that the motor cortex (MTC) and the ACC (layer I-III electrode) field-potential (FP) slow wave oscillation power parallel one another on the pre-conditioning day (when their data is normalized to their average slow delta oscillation power across 8 hours). However, after the animals were trace fear conditioned, the slow delta oscillation power significantly increased in the ACC over that of the motor cortex (* is *p *< 0.05 compared to motor cortex post-conditioning). (B) Example of the slow delta oscillation during NREM sleep, recorded in the motor cortex (MTC) and the ACC before and after trace fear conditioning. When the mouse goes to sleep there is an increase in slow delta oscillation power in the EEG of the ACC with only a modest increase in the motor cortex. (C) Electrodes in the ACC that did not straddle layers I-III did not show and increase in slow delta oscillation power after trace fear conditioning. Indeed the motor cortex (MTC) served as a poor control for the ACC in these cases. This can be seen during the pre-conditioning session, in which the normalized slow wave oscillation power of the ACC and MTC activity did not overlap.

**Figure 10:**
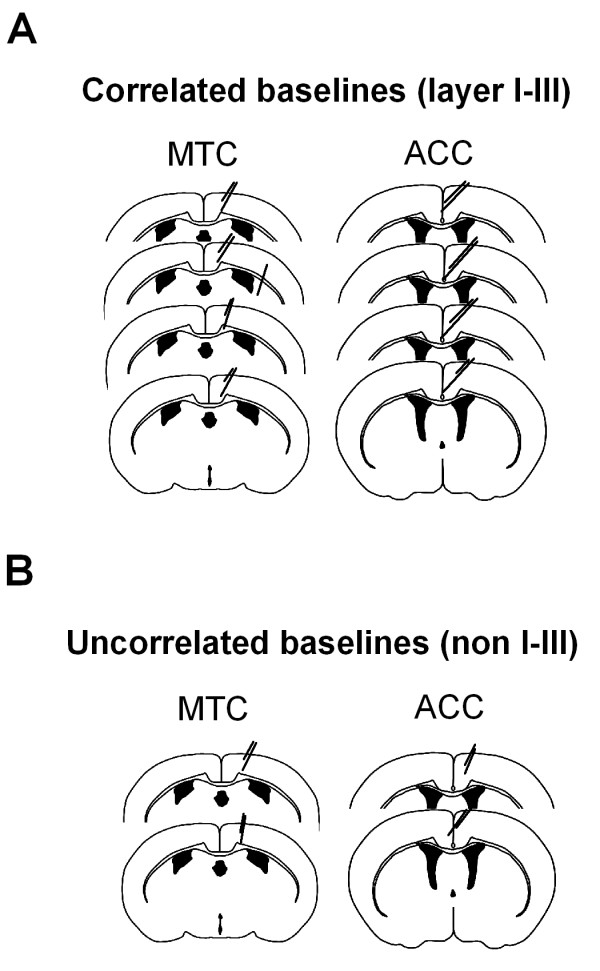
**Position of electrodes**. Position of electrodes, in the motor cortex (MTC) and the ACC, from experiments in figure 9. Data from figure 9 was grouped according to whether the electrodes were located within layers I-III of the ACC or outside this region (non I-III). If electrodes were in layers I-III, the normalized baseline sleep correlated with that of the motor cortex (Indicated as correlated or uncorrelated).

Simultaneous bipolar intracortical field recordings were performed in the ACC and the motor cortex of WT mice. The motor cortex served as a control for the ACC to examine regionally selective slow oscillation enhancements. The behavioral paradigm for this second set of experiments is identical to that outlined in Figure 1 (however a 15 sec trace interval was used). We first standardized the location of the recordings from the ACC to carry out this examination (Figure 8). This experiment revealed that optimal placement of the bipolar electrodes in the ACC was that straddling layers I-III, consistent with others [42]. This recording configuration was set up so as to minimize volume conducted theta rhythms from the hippocampus and 60 Hz noise from the external environment. Data was normalized for the purposes of comparison between motor cortex and the ACC. This was necessary as the voltage of the motor cortex was often much larger than that recorded from the ACC. During sleep, in the recording environment, the normalized slow delta oscillation power of the motor cortex and ACC mirrored one another across the 8 hour recording period (Figures 9A and 9B). Following trace fear conditioning, there was a significant difference between the ACC and motor cortex (*F*_(1,6) _= 6.37; *p *= 0.045, two-way ANOVA-RM). Post-hoc analysis revealed that there was an enhancement in slow oscillation power in the ACC above that of the motor cortex (Figures 9A and 9B, *t *= 2.52; *p *= 0.045). In two cases, where bipolar electrodes did not straddle layers I-III (Figure 10 for electrode positions), the ACC slow delta oscillation power did not mirror that of the motor cortex during pre-conditioning sleep (Figure 9C), and so were not used to examine regional field potential enhancements. Collectively, the result indicates that trace fear conditioning selectively activates brain regions of the ACC in layers I-III during subsequent NREM sleep.

### Learning and 4-7.5 Hz rhythms are correlated with trace fear memory formation

**Figure 11:**
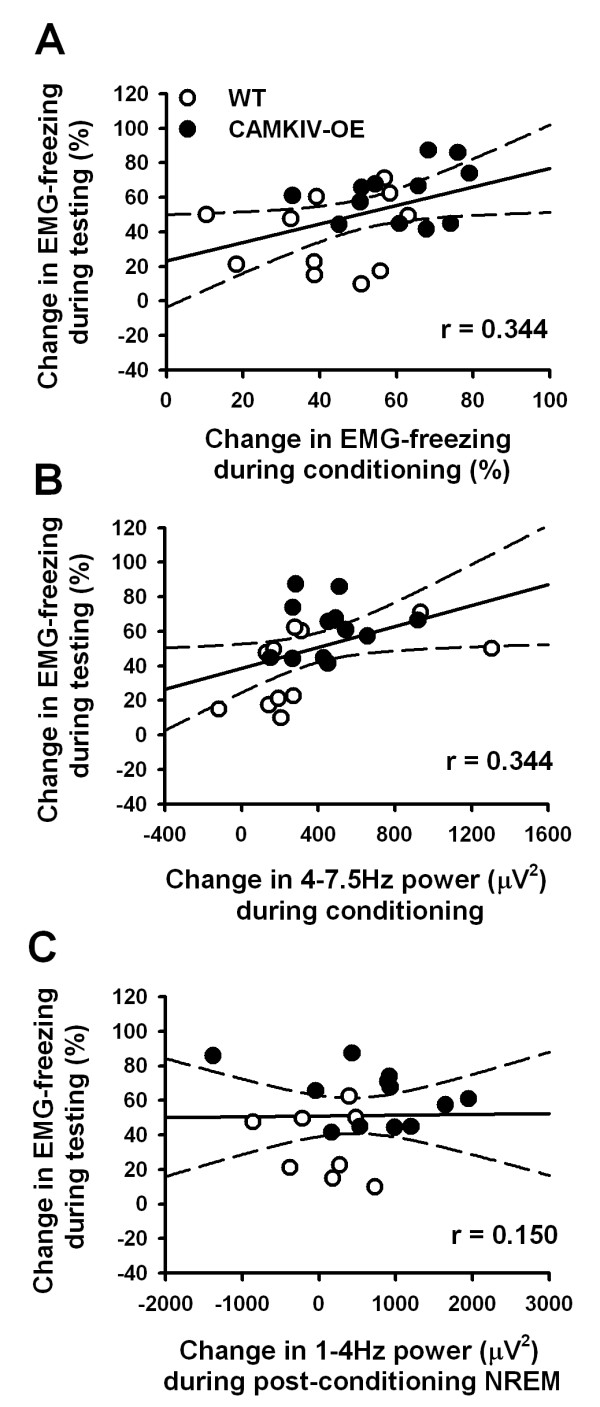
**Variations in learning and 4-7.5 Hz oscillation power are related to memory formation**. (A) The average change in freezing during the trace interval was positively correlated with the average change in fear memory on the testing day. (B) The average change in 4-7.5 Hz power during the trace interval was positively correlated with the average change in fear memory on the testing day. (C) The average change in slow delta oscillation power during post-conditioning sleep was not positively correlated with the average change in fear memory on the testing day. Dashed lines are 95% confidence intervals.

We first examined whether measures of fear conditioning were related to the formation of trace fear memory. Data from CaMKIV over-expressed and WT animals were grouped together. A statistically significant positive correlation was found between the total time spent freezing during the trace interval and the total time spent freezing during the trace interval of the testing day (r^2 ^= 0.188, *p *< 0.039, Figure 11A). Interestingly, a significant positive correlation was found between the percent increase in 4-7.5 Hz power during the trace interval and the total time spent freezing during the trace interval of the testing day (r^2 ^= 0.188, *p *< 0.039, Figure 11B). The result indicates that some degree of variability in 4-7.5 Hz power during the trace interval accounts for a degree of variability in memory encoding. However, in spite of the enhanced slow wave activity in CaMKIV over-expressed mice during the first hour of post-conditioning sleep, no correlation was detected between the percent increases in slow wave activity and the fear memory the following day (r^2 ^= 0.0215, *p *< 0.538, Figure 10C). The result suggests that for the trace fear paradigm, the most important factors controlling the variability of the memory is the degree of freezing and the change in theta oscillation power during conditioning.

## Discussion

Our findings provide the first direct genetic evidence that enhancing CaMKIV expression in the forebrain enhances 4-7.5 Hz oscillations during trace fear conditioning and boosts slow delta oscillations during NREM sleep. We also show that accentuation of trace fear learning, through reduction of the trace fear interval, produces increases in oscillation power during both learning and subsequent sleep. This enhancement could be localized to the frontal cortex which is also likely to be the origin of the enhancements seen in CaMKIV over-expressed mice. Finally, correlation analyses implicate 4-7.5 Hz oscillations in both the learning of the trace fear paradigm and the memory of the conditioned response 24 hours later.

### CaMKIV and natural sleep

CaMKIV mRNA is up-regulated following sleep [35], and CREB knockout mice [53] have reduced levels of arousal, spending more time in NREM sleep. Thus, it could be predicted that CaMKIV over-expression may also impact behavioral states of arousal and sleep. However, unlike CREB knockout mice, CaMKIV over-expression did not alter the amount of time spent in any behavioral state. When EEG power was quantified in the recording environment, CaMKIV over-expressed mice showed an increase in slow delta oscillation power values, which persisted across the first 8 hours of the light period. There are three simple interpretations for this finding; firstly, the enhancement may be related to processing in the novel recording environment, which then leads to and increase in slow delta oscillation power during sleep. Indeed, we did find and increase in 7.5-13 Hz power during wakefulness when animal was exposed to the novel fear conditioning chamber (Figure 4D). Interestingly, this is the same frequency that was decreased in CREB knockout during REM sleep [53]. Consistently, preliminary studies in our lab suggest that when a 4 day acclimatization period (in which animals are hooked to the recording device in the recording environment) is given to CaMKIV mice; the slow wave enhancements during baseline are not present. Future studies, using sample sizes comparable to those used in current study, will need to confirm this possibility. Alternatively, reduced slow wave sleep is thought to correlate with both ageing and memory impairment [54-56], [for review, see [57]]. Since CaMKIV over-expression has been found to rescue memory impairment in aged mice [32], the enhanced slow wave delta power in the recording environment may reflect some degree of rescue of age-dependent slow wave oscillations. Finally, CaMKIV over-expressed mice may have an alteration in their sleep homeostat. However, if this were the case it may be expected that these mice would sleep longer or more frequently during baseline pre-conditioning sleep recordings, which they did not (Table 1). Indeed, our preliminary studies (unpublished) suggest that there is no obvious difference in slow wave sleep recovery as a consequence of 4 hours of sleep deprivation, induced with novel objects and gentle handling in CaMKIV mice.

### CaMKIV and trace fear conditioning

We have previously shown that CaMKIV over-expressed mice have enhancement in trace fear learning and memory [33]. We replicated this finding, utilizing a new EMG-based scoring methodology [38] which permitted evaluation of the mouse behavior in low light condition, during their natural wake cycle. In parallel with trace fear conditioning curves, we found an enhancement in 4-7.5 Hz power, which could be localized to the prelimbic cortex. There is evidence that these prefrontal 4-7.5 Hz rhythms, which exist in mice, rats, monkeys and humans, are involved in attention and learning processes [1-4]. Indeed, trace fear conditioning requires prefrontal cortical structures and sustained attention for learning to take place [49,52,58-60]. Thus we interpret this finding as an increase in attention to, or recall of, the impending shock stimulus in the trace fear paradigm. Indeed this data is consistent with the enhanced LTP observed in the prefrontal region of CaMKIV over-expressed mice [33].

While the hippocampus is known to be a fundamental structure exhibiting ~8 Hz rhythms [for review, see [44]], we suspect that that the prefrontal 4-7.5 Hz enhancement observed during the trace interval does not necessarily correlate with the typical 8 Hz CA1 rhythms. Moreover, we do not think that the 4-7.5 Hz signal recorded during the trace interval was volume conducted from the hippocampus because; the bipolar recording electrodes were placed close together (0.3 to 0.5 mm tip offset) so as to subtract common mode signals [42]. Since the hippocampal theta would be volume conducted from a distance, the signal would be presumed to reach the bipolar electrodes equally. This common signal would then be subtracted out by the inverting and non-inverting inputs of our amplifier. Thus we don't expect a meaningful level of volume conduction is a factor. Finally, cells demonstrating theta modulation have been detected in the prelimbic region of the rat prefrontal cortex in response to spatial learning tasks [3,47,48]. Thus, our results support the notions that the prefrontal 4-7.5 Hz rhythms detected here originate in the prefrontal cortex.

To examine the degree to which the 4-7.5 Hz oscillations were related to learning, correlation analysis were performed. A significant correlation was found between theta power enhancements and learning, confirming that this brain wave frequency was indeed related to the freezing behavior. Since, both freezing behavior and 4-7.5 Hz power demonstrate a learning curve; it is reasonable to suggest that this 4-7.5 Hz frequency reflects memory recall. Indeed, this is supported by the observation that these oscillations were localized to the trace fear interval and not the shock period (Figure 4C).

### CaMKIV over-expression enhances sleep after learning

Various studies have suggested that slow oscillations during sleep are related to memory [5-8]. Moreover, brain derived neurotrophic factor (BDNF) injection in the cortex was found to enhance local slow wave EEG activity in rats [61]. There is also evidence that slow delta oscillations are a consequence of GluR1 phoshorylation [6] or enhanced extracellular glutamate [62], possibly as a consequence of learning during the day. We show that trace fear conditioning, which is enhanced by CaMKIV over-expression, results in concomitant slow delta oscillation enhancement in the cortex. Thus, it appears that CaMKIV, a key upstream molecule which phosphorylates CREB [26], can modulate the expression of slow delta oscillations as a consequence of learning. Interestingly, slow delta oscillation enhancements could be localized to the ACC, predominating in layer I-III. Consistently, removal of the anterior cingulate cortex impairs trace fear, but not delay fear conditioning [52]. In addition, inactivation of the ACC, days after fear training, blocks fear memory retrieval [63]. Also, it has recently been reported that neurons of the prefrontal cortex consolidate trace eye-blink memory days after conditioning [64]. Thus CaMKIV mediated phosphorylation of CREB may be a major mediator of this consolidation, in part through enhancement of local slow delta oscillations during NREM sleep.

It is possible that differences in post-conditioning sleep expression may be related to differences in arousal during the training period, and not brain plasticity. However we did not observe any increase in CaMKIV mice above that of WT mice in the high frequency EEG range during the training trials (for frequencies greater than 13 Hz). The most prominent change was an increase in 4-7.5 Hz EEG power and a decrease in 7.5-13 Hz power (Figure 4) relative to WT. Indeed all EEG responses to pain stimuli were identical between CaMKIV over-expressed and WT mice. Moreover we have previously shown that CaMKIV over-expressed mice have normal responses to acute pain [33]. Finally, we show that the slow wave enhancement have some regional specificity to the anterior cingulate cortex when compared to the motor cortex.

### Variations in learning and 4-7.5 Hz oscillation power are related to memory formation

We found that CaMKIV over-expression enhances fear memory when studied ~24 hours after training. While the elevations in 4-7.5 Hz power during conditioning and slow delta power (1-4 Hz) during NREM sleep are seen in CaMKIV mice, suggestive of learning and memory processes, the data remain merely suggestive. Thus, it was necessary to examine whether the magnitude of changes in learning, 4-7.5 Hz oscillations, and slow wave delta power actually correlated with the magnitude of memory formation. To demonstrate that this was a valid test, we first showed that learning and memory were correlated. We next demonstrated that the change in 4-7.5 Hz power during learning, but not 1-4 Hz during post-training sleep, was significantly correlated with subsequent memory. Based on the findings we suggest that the best predictors of memory formation, at least for the trace fear paradigm, are the degree of freezing and change in 4-7.5 Hz power during conditioning. This should draw attention to the importance of encoding information during wakefulness. While studies report benefits on of NREM sleep [5,7,8,65] on memory encoding, the importance of memory consolidation during wakefulness need not be overlooked. Indeed a few studies have suggested that neural replay of past events even occurs in wakefulness [13,14].

The current study does not discriminate whether the observed oscillations during the trace interval reflect a point of memory consolidation, recall or attention. However experiments examining post-training sleep oscillations suffer from the same confound, for which there is no direct behavioral correlate. Unit recording of neurons which demonstrate learning, combined with local pharmacological manipulations of relevant plasticity pathways, may help resolve this issue. Taken together, studies which examine the interactions of sleep and memory may benefit from examining brain waves changes occurring during wakefulness in addition to brain wave changes during sleep.

The current study did not utilize a non-associative control. Rather it is shown that direct manipulation of learning and memory through transgenic means, produces corresponding enhancements in brain wave oscillations in wakefulness and in sleep. Moreover, correlation analyses indicate the degree to which measures of brain wave oscillations power, during the trace interval, is predictive over fear learning and memory. However the low r^2 ^values for all of our correlations suggest that some degree of freezing behavior during the conditioning phase may not be related to learning, but may be related to the more general process of fear itself. Thus, the use of a non-associative control may be of helpful in future paradigms to more clearly discriminate learning from the more general behavior of freezing.

One difference between the current study and other studies of learning and NREM sleep; is that our paradigm impacts the stress levels of an animal, which may counteract the depth of sleep. Thus we may be measuring two processes, one of stress induced sleep prevention and the other of sleep potentiation. Indeed, this is why using motor cortex control electrodes is ideal (Figure 9B). In such a situation, the null hypothesis would be that both regions of cortex will respond similarly to trace fear conditioning. Irrespectively, we found that slow wave activity in the ACC was potentiated above that of the motor cortex. Thus, the slow delta oscillation power enhancements seen in this study may be related to either learning or memory, or even a build up of extracellular glutamate left in the synaptic cleft from the day's events [62]. Future experiments will have to clarify it precise role.

It has been previously shown that CaMKII is up-regulated following periods of wakefulness [6]. Thus, similar to CaMKIV, it may be expected that over-expression of CaMKII would also potentiate brain waves. Enhancement of 4-7.5 Hz or slow 1-4 Hz oscillations may occur through AMPA receptor insertion or phosphorylation of networks which are being triggered by a common source (e.g. hippocampus or thalamus). Interestingly, it has recently been shown that local knock down of CaV1.2 channels in the ACC can block fear learning and this same region also exhibits theta rhythms during fear learning [66]. This finding is consistent with the current observations of theta rhythms in the prelimbic cortex and slow wave potentiation in the ACC. Finally, it has been suggested that T-type calcium channels may also play a role in sleep-dependent memory consolidation, either through long-term synaptic depression or potentiation [67]. However, this line of investigation is still in its infancy and will require more specific pharmacological compounds [e.g. [68]].

## Abbreviations

ACC: anterior cingulate cortex; CaMKIV: Calcium/calmodulin dependent protein kinase IV; CREB: cAMP response element-binding protein; EEG: electroencephalogram; EMG: electromyogram; Hz: Hertz; REM: rapid-eye-movement; WT: wild type.

## Funding

This work was supported by the EJLB-CIHR Michael Smith Chair in Neurosciences and Mental Health and a CIHR operating grant CIHR84256 to MZ Work was also supported by CGS-NSERC Scholarship to HS

## Competing interests

The authors declare that they have no competing interests.

## Authors' contributions

HS conducted and designed the experiments, analyzed data, and wrote the manuscript. VW contributed to data analysis and histological verification of electrodes. HF and SK developed CaMKIV over-expressing transgenic mice. MZ contributed to important discussions and manuscript writing. All authors read and approved the final manuscript
